# 17q12 Recurrent Deletions and Duplications: Description of a Case Series with Neuropsychiatric Phenotype

**DOI:** 10.3390/genes12111660

**Published:** 2021-10-21

**Authors:** Roberta Milone, Raffaella Tancredi, Angela Cosenza, Anna Rita Ferrari, Roberta Scalise, Giovanni Cioni, Roberta Battini

**Affiliations:** 1 Department of Developmental Neuroscience, IRCCS Fondazione Stella Maris, Calambrone, 56128 Pisa, Italy; rmilone@fsm.unipi.it (R.M.); rtancredi@fsm.unipi.it (R.T.); acosenza@fsm.unipi.it (A.C.); arferrari@fsm.unipi.it (A.R.F.); rscalise@fsm.unipi.it (R.S.); gcioni@fsm.unipi.it (G.C.); 2 Tuscan PhD Program of Neuroscience, University of Florence, Pisa and Siena, 50139 Florence, Italy; 3 Department of Clinical and Experimental Medicine, University of Pisa, 56126 Pisa, Italy

**Keywords:** autism spectrum disorder, intellectual disability, recurrent CNVs, psychiatric disorders, RCAD, epilepsy

## Abstract

Syndromic neurodevelopmental disorders are usually investigated through genetics technologies, within which array comparative genomic hybridization (Array-CGH) is still considered the first-tier clinical diagnostic test. Among recurrent syndromic imbalances, 17q12 deletions and duplications are characterized by neurodevelopmental disorders associated with visceral developmental disorders, although expressive variability is common. Here we describe a case series of 12 patients with 17q12 chromosomal imbalances, in order to expand the phenotypic characterization of these recurrent syndromes whose diagnosis is often underestimated, especially if only mild traits are present. Gene content and genotype-phenotype correlations have been discussed, with special regard to neuropsychiatric features, whose impact often requires etiologic analysis.

## 1. Background

Neurodevelopmental disorders could be considered either idiopathic or syndromic, depending on whether the neuropsychic conditions are isolated or associated with the involvement of malformations [1,2].

Array comparative genomic hybridization (Array-CGH) is still considered the first-tier clinical diagnostic test for individuals with both idiopathic and syndromic neurodevelopmental disorders [3,4,5]. It allows one to detect both sporadic genomic imbalances and recurrent microdeletion/microduplication syndromes [6,7].

Among them, 17q12 deletions and duplications are two reciprocal, recurrent genomic imbalances (i.e., copy number variations, CNVs) characterized by neurodevelopmental disorders (i.e., developmental delay, intellectual disability, autism spectrum disorder, language disorders, and attention deficit/hyperactivity disorder), epilepsy, and structural brain anomalies. Eye abnormalities, visceral developmental disorders (i.e., provoking cardiac, renal, pancreatic, and hepatic anomalies), gastrointestinal tract atresia, genital, and endocrine abnormalities are often included among the main characteristics [8,9].

The recurrence of 17q12 genomic imbalances depends on a susceptible region flanked by segmental duplications, namely low copy repeats (LCRs), prone to nonallelic homologous recombination (NAHR) [10]. The critical region associated with these recurrent syndromic conditions is sized at about 1.4 Mb, and includes 17 refseq genes [8].

Here we describe a large series of patients with 17q12 chromosomal imbalances in order to deepen the highly variable clinical presentation and to expand the phenotypic characterization of these recurrent syndromes, whose diagnosis is often underestimated, especially if only mild features are detected. In addition, we discuss the more significant candidate genes, with special focus on brain-enriched genes.

## 2. Materials and Methods

### 2.1. Patients

The patients were recruited in a 10-year period at IRCCS Stella Maris Foundation. They presented neurodevelopmental-neuropsychiatric disorders which deserved to be followed up after diagnosis. Some of them also presented visceral malformation.

### 2.2. Molecular Analysis

Array-CGH analysis was performed using the Agilent 8 × 60 K microarray oligonucleotide platform with a median resolution of 100 Kbp, according to the manufacturer’s protocol (Agilent Technologies, Santa Clara, CA, USA). The detected CNVs coordinates refer to the Genome Reference Consortium Human Build 37 (GRCh37/hg19). The CNVs were confirmed by quantitative polymerase chain reaction (qPCR). Segregation analyses in parental DNA were performed by qPCR.

According to the guidelines of the Italian Society of Human Genetics (https://www.sigu.net (accessed on 15 April 2021)), each CNV was compared with those from healthy subjects collected in the Database of Genomic Variants (DGV) (http://projects.tcag.ca/variation (accessed on 15 April 2021)). In addition, CNVs were not reported if they were described in at least three independent DGV studies and/or did not involve known genes. As recommended by the American College of Medical Genetics guidelines [11] and the European Guidelines for constitutional cytogenetic analysis [12], we selected pathogenic/likely pathogenic CNVs.

International databases such as Database of Chromosome Imbalance and Phenotype in Humans using Ensembl Resources (DECIPHER) (http://www.sanger.ac.uk/PostGenomics/decipher (accessed on 10 October 2021)), University of California Santa Cruz (UCSC) Genome Browser (https://genome.ucsc.edu/ (accessed on 10 October 2021)), Pubmed (http://www.ncbi.nlm.nih.gov/pubmed (accessed on 10 October 2021)), Online Mendelian Inheritance in Man (OMIM) (http://www.omim.org (accessed on 15 April 2021)), and GeneCards (https://www.genecards.org (accessed on 15 April 2021)) were also consulted for evaluating genotype-phenotype association.

### 2.3. Neuropsychiatric Assessment

Neuropsychiatric evaluation was conducted by a multidisciplinary team with expertise on neurodevelopmental and psychiatric disorders, through clinical observations, speech test batteries, and specific scales (i.e., ADOS 2: Autism Diagnostic Observation Schedule 2; ADI-R: Autism Diagnostic Interview—Revised; Griffiths Scales of Child Development; WPPSI-III: Wechsler Preschool and Primary scale–III edition; WISC-III, WISC-IV: Wechsler Intelligence Scale for Children–III or IV edition; Leiter-R: Leiter International Performance Scale—Revised; CPRS-R: Conners’ Parent Rating Scales—Revised).

## 3. Results

The age of patients ranged from 3 years old to 17 years old. We have found six patients harboring 17q12 microdeletion, whose size ranged from 1.32 Mb to 1.73 Mb, and six patients with 17q12 microduplication, whose size ranged from 0.92 to 1.73 Mb. Four were females and eight were males.

Three pairs of patients had the same genomic imbalances (Patients 4 and 5, Patients 9 and 10, and Patients 11 and 12, respectively), with perfect overlap between the breakpoints, besides Patients 1 and 2 who were siblings and inherited the same 17q12 deletion from their father. Patients 7 and 8 had the same imbalance size, although the former carried a microdeletion and the latter a microduplication. Only Patient 1 had an additional imbalance which involved the 6q22.31 region.

Genetic and neuropsychiatric features of the 12 patients have been summarized in Table 1a (Demographic distribution and genetic findings) and Table 1b (Patients’ phenotypic characterization).

## 4. Discussion

17q12 deletion and duplication syndromes determine an increased risk for neurodevelopmental and neuropsychiatric disorders, such as developmental delay, ID (mild to severe), ASD, psychotic disorder, anxiety, and bipolar disorder [8,9]. They have also been related to visceral and endocrine anomalies, these being the implicated organs likely sensitive to dosage changes of genes located in the 17q12 chromosomal region. However, the prevalence of specific features (i.e., kidney involvement and facial dysmorphisms) in patients with deletions, compared with others (i.e., more severe esophageal defects) in patients with duplications, suggests an organ-specific susceptibility to higher or lower gene dosage [8,9]. In addition, patients with 17q12 duplication display a wide phenotypic spectrum due to reduced penetrance and variable expressivity, ranging from relevant cognitive impairment to asymptomatic carriers. Consequently, the prevalence of this genomic imbalance is expected to be underestimated [13], and more frequently inherited in respect to 17q12 deletion [9].

Microcephaly has been more often associated with 17q12 duplication [13], while macrocephaly has been associated with the reciprocal chromosomal imbalance [8]. In our cohort of patients nobody had altered occipito-frontal circumference.

Non-specific brain anomalies have been reported in 17q12 genomic imbalances. These include ventricular dilatation, mild cerebellar atrophy, abnormal signal intensity of subcortical white matter, atrophy of the hippocampus [9], cortical malformations [13], and thinning/agenesia of the corpus callosum [8]. In our patients, brain MRIs were substantially normal, independently from the range of clinical severity. Only Patient 3 showed aspecific peritrigonal white matter anomalies. This evidence may confirm that ultrastructural and functional anomalies, which are determined by the dysregulations of key molecules, underlie neurodevelopmental disorders even without detectable brain structure abnormalities [14,15]. 

Reduced weight at birth and intrauterine growth retardation were noticed in some of our patients. Abnormal prenatal evidences have been reported in patients with genomic imbalance, significantly with 17q12 deletions [16].

By analyzing the critical 17q12 region gene content, *HNF1B* may be responsible for the majority of features related to 17q12 microdeletion/microduplication syndromes, including neurodevelopmental disorders, this gene being widely distributed [17]. In particular, this gene confers the acronym RCAD (renal cysts and diabetes) to the 17q12 deletion syndrome, being responsible for the combination of kidney dysfunction and maturity-onset diabetes of the young (MODY5) [9]. Kidney alterations were present in two patients, although abdominal ultrasounds were executed only in five patients. No patient had diabetes, yet it is not possible to exclude its future manifestation, since the studied cohort is only composed of youngsters.

*LHX1* is involved in neuronal differentiation, axon migration, and in cerebellum development [18,19], thus it may concur with ASD and with other neurodevelopmental disorders described in these recurrent syndromes [14,20,21].

Recently, Gametogenetin Binding Protein 2 (*GGNBP2*) has been included among ASD candidate genes and reported in the Autism Informatics Portal AUTDB (http://autism.mindspec.org/autdb (accessed on 10 October 2021), although at the time of writing it has not been associated with any Mendelian disease [22]. Interestingly, this gene is co-expressed with other autism- and brain-enriched genes such as *MECP2* [22], suggesting a role in pathways implicated in the ASD pathology.

We could also hypothesize that *SYNRG*, a gene involved in Golgi trafficking and, in particular, in inwardly rectifying K+ channels (e.g., Kir4.1) export, may concur with ASD [23], and even be associated with epilepsy [24].

*TADA2A* acts as a transcriptional activator, managing histone acetylation and chromatin organization. Therefore, it may regulate gene expression in critical development phases [25]. On the other hand, *AATF* inhibits the histone deacetylase HDAC1 and activates cell cycle progression [25]. Both genes may thus influence neurodevelopment.

Patient 1 had the most severe picture, considering both his neuropsychiatric profile and multiorgan involvement. Besides 17q12 microdeletion inherited from his father and shared with his sister, he carried an additional 6q22.31 duplication inherited from his mother. This duplication encompassed *TBC1D32* without a definite pathogenic role, although this gene is involved in cell cycle and consequently in early development, particularly in sonic hedgehog signaling. It has been related to ciliopathies and is a brain-enriched coding gene [26]. Therefore, we cannot exclude the possibility that this double hit may concur to aggravate the composite picture of Patient 1, as described in other cases [27]. Another hypothesis regards incomplete penetrance and variable expressivity of 17q12 genomic imbalances, reported in many dominant mechanisms [28]. Indeed, his peculiar ocular picture could be attributable to 17q12 deletion, since eye abnormalities, such as strabismus and horizontal nystagmus, are included within its features [9]. Furthermore, his paroxysmal movement disorder may show the contribution of at least two genes included in the 17q12 region, namely the aforementioned *SYNRG* and *MYO19*. The former may alter calcium ion binding by regulating the clathrin-mediated vesicular traffic and concurring to cause channelopathy [29] in a similar way to what has been described in relation to Kir channels [23]. The latter is a mitochondrial gene implied in cytokinesis and in reactive oxygen species (ROS) distribution [30], and could resemble other genes’ functionality, such as myofibrillogenesis regulator 1, itself associated with paroxysmal movement disorders [31].

In Patients 7 and 8, the genomic imbalances were perfectly overlapping, although reciprocal (i.e., the former had a deletion and the latter a duplication). Their dimension was broader than the others described in our sample, and they included four genes encoding chemokines, such as *CCL1L1*, *CCL3L3*, *CCL4L1*, and *CCL4L2*, besides the genes of the 17q12 critical region. Differing from the majority of the other patients, who presented ASD and intellectual disability, their clinical picture was characterized by ADHD, anxiety, and mood disorder, associated with cognitive impairment. Recent studies have also emphasized the role of neuroinflammation in these neurodevelopmental and psychiatric disorders [32,33,34].

Furthermore, Patient 7 had a cryptogenic cholangiopathy which may be related to 17q12 microdeletion, since elevated hepatic transaminase enzyme levels, cholestasis, liver abnormalities involving choledochal, and bile duct cysts have been reported [9]. We cannot exclude the possibility that chemokines involvement in this broader deletion may have also contributed to this hepatic disorder, which often has an inflammatory origin [35].

Patients 9 and 10 had an overlapping 17q12 deletion and a similar clinical picture, both presenting with high-functioning ASD and receptive-expressive language disorder.

Interestingly, Patients 11 and 12, who presented a smaller duplication outside and upstream to the critical 17q12 duplication syndrome, met the diagnostic criteria for ASD similar to patients with 17q12 duplication syndrome, although different genes are involved. A fascinating hypothesis regards the immunologic imbalance, which has been related to ASD, deriving from these CNVs which shared the same distal breakpoints and the same gene content, even though the proximal breakpoints were different. Specifically, cytokine and chemokine profiles alteration, which is derived from *CCL2* [36], *CCL7*, and *CCL11* [37] imbalance in our patients, may provoke this neurodevelopmental disorder. Furthermore, *ASIC2* has been linked with gut microbiome modulation [38], which is nowadays considered responsible for many psychiatric disturbances, including ASD, since it may contribute to inflammation via the gut–brain axis [39]. Autistic regression has recently been related to neuroinflammation [40], as occurred in Patient 11, who had a cessation of language development and a regression of relational abilities after a respiratory infection, and each subsequent inflammatory episode connoted similar functional consequences. *ASIC2* is also involved in neurotransmission [41]. Neural adhesion and immunologic functions may also be influenced by *TMEM132E* [42], which has been associated with panic disorder, while other TMEM family members have been related to high-functioning autism [43]. 

In the light of this evidence, we may hypothesize as to their implication in human pathology, although both duplications are reported to be frequent in the DGV.

A confirmation of 17q12 recurrent CNVs’ strong association with neurodevelopmental disorders has been recently highlighted considering brain-enriched coding and long non-coding (lnc) RNA genes [44]. In particular, authors reported two patients: the former carried a duplication sizing 40 Mb not involving brain-enriched genes but encompassing five lncRNAs; the latter carried a deletion sizing 1.410 Mb involving four brain-enriched genes and five lncRNAs. They shared the same proximal breakpoints. In the Decipher databases no patients have been reported with CNVs overlapping with the duplication, while several patients have been reported with the same deletion, many of whom present ID, one presenting with intrauterine growth retardation (IUGR) and hyperechogenic kidneys, and one presenting with diabetes. In our series of patients, ten had proximal breakpoints downstream to those presented in [44], although the brain-enriched coding and lncRNA genes are the same (respectively, four and five, Table 2, [44]). IUGR was present in one patient with deletion and in another with duplication. The other two patients carrying smaller CNVs upstream to the critical region presented two brain-enriched coding and three lnc RNA genes (Table 2). While the role of brain-enriched coding genes is sufficiently known in 17q12 imbalances as described above, understanding epigenetic regulation due to lncRNAs during cell differentiation and development is continuously ongoing. LncRNAs are involved in epigenetic reprogramming during cell growth and development by acting as chromatin, transcriptional, post-transcriptional, and post-translational regulators [45]. For this reason, lncRNAs dysregulation is related to several neurodevelopmental disorders, since they interfere with synaptic transport/signaling and long-term potentiation and depression [45].

In our series, three pairs of patients displayed overlapping genomic imbalances and also shared overlapping phenotypes; therefore, genotype-phenotype correlations may appear to be more easily interpreted. Furthermore, as evidenced by Patients 7 and 8, 17q12 deletions and duplications could provoke similar neurodevelopmental disorders, although congenital malformations are more frequent in the former [8].

One limitation of our study regards the lack of functional studies which could permit one to better understand gene expression, also regulated by lncRNAs. Furthermore, prediction and prioritization of bioinformatic studies may help in predicting transcript sequence features and performing functional characterization of the candidate genes and lncRNAs [46]. 

## 5. Conclusions

Syndromic neurodevelopmental disorders are conditions characterized by the co-occurrence of phenotypic-behavioral anomalies associated with dysmorphic or malformative pictures. Based on the prevalence of the neuropsychiatric or pediatric impact, they usually draw the attention of a specialist who should vet each feature of the child, and examine in depth the biological, functional, and genetic aspects to increase the probability of reaching an etiological diagnosis.

The knowledge evolution about recurrent genomic imbalances and their gene content, with special regard to candidate genes in neurodevelopmental disorders and brain-enriched genes, will allow one to progressively clarify the genotype expression.

## Figures and Tables

**Table 1:** Genetic and neuropsychiatric features of the 12 patients. (**a**): Demographic distribution and genetic findings. (**b**): Patients’ phenotypic characterization.

(a)								
Case	Gender	Pre-Perinatal Parameters and Information	Age	Chromosomal Coordinates (GRCh37-hg 19)	Type of CNV	Size	Segregation	Family History
1	M	W: 2500 g L: 48 cm OFC: 33 cm Apgar scores: 8 (1′), 10 (5′)	15	Chr6:121396856-121528927 Chr17:34816256-36244359	Dup Del	132 Kb 1.43 Mb	Mat Pat	Congenital nystagmus, epilepsy, polycystic kidney, and chronic renal failure in paternal line
2 (sister of case 1)	F	W: 3650 g L: 50 cm OFC: 30 cm Apgar scores: 8 (1′), 9 (5′)	8	Chr17:34816256-36244359	Del	1.43 Mb	Pat	Congenital nystagmus, epilepsy, polycystic kidney, and chronic renal failure in paternal line
3	M	Pregnancy complicated by threats of preterm labor and oligohydramnios Birth parameters: NA	8	Chr17:34851337-36168245	Del	1.32 Mb	De novo	NA
4	M	Pregnancy complicated by IUGR W: 2500 g L: 46 cm OFC: NA Apgar scores: NA	5	Chr17:34851537-36168104	Del	1.32 Mb	De novo	Language delay and depressive mood in maternal line; epilepsy in paternal line
5	F	W: 2900 g L: 49 cm OFC: 35 cm Apgar scores: 8 (1′), 9 (5′)	3	Chr17:34851537-36168104	Del	1.32 Mb	NA	NA
6	M	W: 2990 g L: 49 cm OFC: NA Apgar scores: NA	7	Chr17:34817422-36079369	Dup	1.27 Mb	Pat	Language disorders, strokes, and autoimmunity disorders (i.e., multiple sclerosis) in paternal line
7	M	W: 2550 g L: NA OFC: NA Apgar scores: NA Jaundice since the first months of life	15	Chr17:34437475-36168104	Del	1.73 Mb	NA	Negative
8	F	Pregnancy complicated by IUGR and maternal gestosis. Delivery at the 31st GW by urgent cesarean section W: 1100 g L: 38 cm OFC: 25.5 cm Apgar scores: 7 (1′), 10 (5′) Post-natal growth <5th centile.	17	Chr17:34437475-36168104	Dup	1.73 Mb	NA	Hashimoto thyroiditis and bipolar disorder in maternal line; renal insufficiency, learning disability, and anxiety disorder in paternal line
9	M	NA	6	Chr17:34817422-36168104	Dup	1.35 Mb	NA	NA
10	M	Pregnancy complicated by gestosis and cardioaspirine assumption Emergency cesarean section at term Cardio-respiratory depression Resuscitation maneuvers W: 3000 g L: 50 cm OFC: 32 cm Apgar scores: 0 (1′), 7 (5′), 9 (10′)	5	Chr17:34817422-36168104	Dup	1.35 Mb	NA	Positive for mood and anxiety disorders (not specified in which line)
11	M	NA	3	Chr17:32007395-32922965	Dup	0.92 Mb	NA	NA
12	F	NA	7	Chr17:31953228-32922965	Dup	0.97 Mb	Mat	NA
** (b) **								
** Case **		** Instrumental Exams **		** Neuropsychiatric Tests **	** Neuropsychiatric Features **			** Drug Therapy **
1		Ophthalmologic evaluation: mild parapapillary dystrophy prevailing in the left eye due to the high myopic grade. Echocardiography: mild pulmonary valve insufficiency. Abdominal ultrasound: N EEG: N Brain MRI: N *FMR1* analysis: N		Clinical observation	DD, ASD, severe ID, nystagmus, esotropia, paroxysmal movement disorder, aggressive behavior, hyperactivity, mood dysregulation (hypothymia, somnolence, and psychomotor slow-down VS euphoria, excitation, and incongruous laughing), stereotypies, sensorial stimulations			RIS, VPA, CBZ
2 (sister of case 1)		Abdominal ultrasound: right renal hypoplasia. EEG: paroxysmal anomalies at the vertex and on the right occipital-parietal-posterior temporal area, which diffused to the contralateral hemisphere and were activated by sleep. Brain MRI: N		WPPSI-III Speech test battery Clinical observation	DD, Mild ID, epilepsy, selective mutism			PB
3		Abdominal ultrasound: congenital bilateral kidney malformation (i.e., hypo-dysplasia). Brain MRI: non-specific hyperintensity of peritrigonal white matter.		WISC-IV Speech test battery Clinical observation	DD, Mild ID, receptive-expressive LD, ODD			none
4		Audiological evaluation: N EEG: N Metabolic work-up: N Standard karyotype: N *FMR1* analysis: N		ADOS 2 Module 1: positive ADI-R: positive Clinical observation	DD, ASD			none
5		Audiological evaluation: N Metabolic work-up: N Brain MRI: N EEG: N		ADOS-2 Module 1: positive, severe symptoms Clinical observation	ASD, DD			none
6		Abdominal ultrasounds: N Ophthalmological evaluation: N Brain MRI: low cerebellar amygdalae. Neurometabolic work-up: N *FOXP2* analysis: N		WPPSI-III Speech test battery Clinical observation	Verbal dyspraxia, receptive-expressive LD, normal non-verbal cognitive level			none
7		Biliary acids dosage, transaminases dosage, γ-glutamyl transferase dosage, cholesterol and triglycerides dosage: elevation, indicating intrahepatic cholangiopathy. Brain MRI: N EEG: N Abdominal ultrasound: N		WISC-IV: VCI 66, WMI 64, PRI 93, SPI 91. Speech test battery Clinical observation	Mild ID, depressive mood, attention deficit, behavior disorder, deficient in communicative and social skills			Ursodeoxycholic acid
8		Transfontanellar ultrasound: N Orthopedic evaluation: hip dysplasia, tibia vara and talus-valgus feet. Brain MRI: N		WISC-III: TIQ 73, VIQ 69, PIQ 85. Psychiatric evaluation	BCI, ADHD type sluggish cognitive tempo, cyclothymic disorder, anxiety disorder, thought disorder			GAB
9		NA		Leiter-R ADOS-2 Module 1: positive Speech test battery Clinical observation	ASD, non-verbal cognitive level at lower limits of the norm, receptive-expressive LD, verbal dyspraxia			none
10		Brain MRI: low cerebellar amygdalae. Metabolic work-up: N *FMR1* analysis: N		Leiter-R ADOS 2 Module 1: positive, high level of symptoms ADI-R: positive Clinical observation	ASD, normal non-verbal cognitive level, regulation disorder with motor instability, attention deficit, irritability Requestive verbal productions, non-communicative jargon productions and echolalia Hearing, tactile, oral, and olfactory hypersensitivity, visual stimulation research, aggression due to low frustration tolerance			none
11		Audiological evaluation: N EEG: N ECG: N Neurometabolic work-up: N *FMR1* analysis: N		ADOS-2 Module 1 Griffiths scales Clinical observation	Language delay, periodic relational and communicative regression after inflammatory events alternated with temporary recovery, ASD, sensorial hyposensitivity			none
12		Brain MRI: N Neurometabolic work-up: N *FMR1* analysis: N		Leiter-R: IQ 82 ADOS-2 Module 2: positive ADI-R: positive CPRS-R Clinical observation	ASD, receptive-expressive LD, ADHD, anxious traits, BCI			none

**Legend:** (**a**): M: male; F: female; W: weight; L: length; OFC: occipito-frontal circumference; GW: gestational week; NA: not available; IUGR: intrauterine growth retardation; CNV: copy number variation; Del: deletion; Dup: duplication; Pat: paternal; Mat: maternal; (**b**): EEG: Electroencephalogram; MRI: Magnetic Resonance Imaging; ECG: electrocardiogram; N: Normal; ADOS 2: Autism Diagnostic Observation Schedule 2; ADI-R: Autism Diagnostic Interview—Revised; WISC-III, WISC-IV: Wechsler Intelligence Scale for Children—III or IV edition; Leiter-R: Leiter International Performance Scale—Revised; TIQ: Total Intelligence Quotient; VIQ: Verbal Intelligence Quotient; PIQ: Performance Intelligence Quotient; CPRS-R: Conners’ Parent Rating Scales—Revised; VCI: Verbal Comprehension Index; WMI: Working Memory Index; PRI: Perceptual Reasoning Index; SPI: Speed Processing Index; ASD: Autism Spectrum Disorder; ID: Intellectual Disability; VS: versus; ADHD: Attention Deficit Hyperactivity Disorder; BCI: Borderline Cognitive Impairment; LD: Language delay; ODD: Oppositional-Defiant Disorder; NA: Not Available; RIS: risperidone, VPA: valproic acid; CBZ: carbamazepine; PB: phenobarbital; GAB: Gabapentin.

**Table 2:** Brain-enriched coding and long non-coding RNA genes in the described case series.

GeneID	Gene Symbol	Gene Class	Chromosome	Start	End	Nervous System Subregion
Patients 1–10						
ENSG00000267785.1	*CTD-3194G12.2*	lncRNA intergenic	17	35014119	35106138	nervous system
ENSG00000255509.2	*RP11-445F12.1*	lncRNA divergent	17	35218935	35295767	metencephalon
ENSG00000132130.7	*LHX1*	coding mRNA	17	35293161	35302027	hindbrain
ENSG00000267306.1	*RP11-333J10.2*	lncRNA sense intronic	17	35325601	35343932	brain
ENSG00000108264.12	*TADA2A*	coding mRNA	17	35766977	35849456	nervous system
ENSG00000006114.11	*SYNRG*	coding mRNA	17	35872195	35969446	nervous system
ENSG00000267542.1	*RP11-697E22.1*	lncRNA divergent	17	35969609	35973886	brain
ENSG00000267668.1	*RP11-697E22.2*	lncRNA divergent	17	36002967	36049795	forebrain
ENSG00000174093.6	*RP11-1407O15.2*	coding mRNA	17	36126188	36413351	nervous system
ENSG00000146350.9	*TBC1D32*	coding mRNA	6	121275124	121655875	nervous system
Patients 11 and 12						
ENSG00000265356.1	*RP11-17M24.1*	lncRNA sense intronic	17	32164881	32311341	telencephalon
CATG00000031141.1	*CATG00000031141.1*	lncRNA antisense	17	32258088	32264624	telencephalon
CATG00000033437.1	*CATG00000033437.1*	lncRNA sense intronic	17	32390758	32396195	nervous system
ENSG00000197322.1	*C17orf102*	coding mRNA	17	32901142	32906388	nervous system
ENSG00000181291.5	*TMEM132E*	coding mRNA	17	32906513	32966579	nervous system

**Legend:** lncRNA: long non-coding RNA; mRNA: messenger RNA.

## Data Availability

Publicly available datasets were analyzed in this study. This data can be found at: https://decipher.sanger.ac.uk/ (accessed on 15 April 2021); https://www.genome.ucsc.edu/ (accessed on 10 October 2021); https://www.genecards.org/ (accessed on 15 April 2021); https://omim.org/ (accessed on 15 April 2021).
